# Intradermal Vaccination with PLGA Nanoparticles via Dissolving Microneedles and Classical Injection Needles

**DOI:** 10.1007/s11095-024-03665-7

**Published:** 2024-02-08

**Authors:** Jihui Lee, Malene A. Neustrup, Bram Slütter, Conor O’Mahony, Joke A. Bouwstra, Koen van der Maaden

**Affiliations:** 1 Division of Biotherapeutics, Leiden Academic Centre for Drug Research, Leiden University, 2333CC Leiden, the Netherlands; 2 Tyndall National Institute, Lee Maltings, Prospect Row, Cork, Ireland; 3 Department of Immunology, Leiden University Medical Center, 2300RC Leiden, the Netherlands

**Keywords:** dissolving microneedle, intradermal drug delivery, nanoparticles, vaccine formulation, vaccine immunogenicity

## Abstract

**Purpose:**

A dissolving microneedle array (dMNA) is a vaccine delivery device with several advantages over conventional needles. By incorporating particulate adjuvants in the form of poly(D,L-lactic-co-glycolic acid) (PLGA) nanoparticles (NPs) into the dMNA, the immune response against the antigen might be enhanced. This study aimed to prepare PLGA-NP-loaded dMNA and to compare T-cell responses induced by either intradermally injected aqueous-PLGA-NP formulation or PLGA-NP-loaded dMNA in mice.

**Methods:**

PLGA NPs were prepared with microfluidics, and their physicochemical characteristics with regard to encapsulation efficiencies of ovalbumin (OVA) and CpG oligonucleotide (CpG), zeta potentials, polydispersity indexes, and sizes were analysed. PLGA NPs incorporated dMNA was produced with three different dMNA formulations by using the centrifugation method, and the integrity of PLGA NPs in dMNAs was evaluated. The immunogenicity was evaluated in mice by comparing the T-cell responses induced by dMNA and aqueous formulations containing ovalbumin and CpG (OVA/CpG) with and without PLGA NP.

**Results:**

Prepared PLGA NPs had a size of around 100 nm. The dMNA formulations affected the particle integrity, and the dMNA with poly(vinyl alcohol) (PVA) showed almost no aggregation of PLGA NPs. The PLGA:PVA weight ratio of 1:9 resulted in 100% of penetration efficiency and the fastest dissolution in ex-vivo human skin (< 30 min). The aqueous formulation with soluble OVA/CpG and the aqueous-PLGA-NP formulation with OVA/CpG induced the highest CD4 + T-cell responses in blood and spleen cells.

**Conclusions:**

PLGA NPs incorporated dMNA was successfully fabricated and the aqueous formulation containing PLGA NPs induce superior CD4^+^ and CD8^+^ T-cell responses.

**Supplementary Information:**

The online version contains supplementary material available at 10.1007/s11095-024-03665-7.

## Introduction

Vaccination is one of the most successful interventions to save lives against infectious diseases. However, for intracellular pathogens (and also for cancers) vaccines often lack efficacy. Vaccines against those diseases require the induction of T cells. To boost the induction of T cells by vaccination, two efficient approaches can be applied, (i) deliver the vaccine into organs that are naturally rich in antigen presenting cells (APCs) and (ii) increase the uptake of the vaccine by APCs using nanoparticles (NPs).

Skin is an excellent organ to deliver T cell vaccines since it has a high population of APCs, such as dendritic cells and macrophages. Therefore, intradermal vaccine delivery can promote stronger immune responses for both T cells and B cells. However, conventional needles demand vaccines in liquid form which required a cold chain for storage. Also, they can bring sharp waste, needlestick injury, needle-phobia, and tissue damage if not used correctly [1–3]. These drawbacks show the need for novel vaccine delivery devices. Therefore, dissolving microneedle array (dMNA) has been introduced as one of the efficient approaches, which could revolutionise the way drugs are delivered. dMNAs are frequently made of biodegradable polymers or sugars, and the microneedles typically have a length below 1000 µm. Once the microneedles are inserted into the skin, they dissolve in the skin and release the loaded antigens. In spite of their limited mechanical strength and expensive production cost due to antigen waste [4], dMNA offers numerous advantages over conventional needles. One of the primary advantages is the improved safety profile [5–7]. Unlike conventional needles, dMNA does not produce sharp waste as they dissolve after insertion so that prevents contamination from reuse [8]. Besides, dMNA reduces pain sensation during administration because it barely reaches the nerves. Lastly, dMNA also has protective efficacy as they frequently brought comparable immune responses to hypodermic needle injection even with lower doses [9, 10].

To trigger an immune response against an antigen, it is often necessary to add adjuvants [11]. Not only the immune response can be enhanced by including adjuvants, but a specific immune response can also be generated [12, 13]. A particulate adjuvant, such as poly (D,L-lactic-*co*-glycolic acid) (PLGA) NPs, is a delivery system [13], provides sustained release, ensures co-delivery of antigen and molecular adjuvant, and increases uptake by dendritic cells [14–16]. PLGA is a biodegradable polymer that is approved by the Food and Drug Administration and the European Medicines Agency for different pharmaceutical applications [17], and PLGA NPs have also been used as delivery systems in subunit vaccines in mice studies [15]. The physicochemical characteristics of the particulate adjuvants, such as size, charge, and rigidity, have been shown to affect immunogenicity [12]. NPs seem to favour Th1 and CD8^+^ T-cell-mediated immune responses, whereas microparticles seem to favour Th2 and B-cell-mediated immune responses [12]. Furthermore, dendritic cells which are key in presenting antigens to T cells, as well as activating them, favour the uptake of particles below 200 nm [18]. While B-cell mediated immune responses have been widely introduced for prophylactic vaccines, newer vaccines focus on Th1 and CD8^+^ T-cell immune responses which are needed to combat multiple intracellular pathogens, such as influenza A and tuberculosis [19].

A key strategy in the formulation of new vaccines is to combine both molecular and particulate adjuvants [20, 21]. A molecular adjuvant, such as the Toll-like receptor (TLR) 9 ligand CpG oligonucleotide (CpG), is an analogue of a pathogen-associated molecular pattern which is recognised by pattern recognition receptors on APCs [22]. The combination of TLR ligands with PLGA particles has generated better immune responses compared to using only one adjuvant [23–25]. Furthermore, subunit vaccines with CpG can induce specific Th1 and CD8^+^ T-cell responses in mice and generate long-term survivability against infectious diseases [26, 27].

Conventional methods for producing PLGA NPs, such as single or double-emulsification-based methods, often require large amounts of solvent, are labour-intensive, and are not highly reproducible [28–30]. Compared to conventional methods, microfluidics offers several advantages, including control over the production process, high efficiency, and reduced costs [29, 31]. Therefore, microfluidics is increasingly being used to produce PLGA NPs [32]. In microfluidics, solvents flow through a micro-system consisting of capillaries and chambers allowing precise manipulation of the fluids [33]. This facilitates continuous operation and the production of size-controlled NPs with a narrow size distribution [34], and the technique could therefore be used to produce PLGA NPs with a size below 200 nm, which is needed for Th1 and CD8^+^ T-cell response.

In this study, we evaluate two approaches to improve T-cell induction via (i) PLGA NPs and (ii) dMNA. We first engineered ovalbumin and CpG (OVA/CpG) encapsulated PLGA NPs, and subsequently loaded them into dMNA. In order to ensure the stability of NPs in dMNA, we screened the most suitable polymer formulation of dMNA among three candidates (polyvinylpyrrolidone (PVP), polyvinyl alcohol (PVA), and trehalose) based on the size and polydispersity indexes (PDI) of PLGA NPs. dMNA was fabricated with the selected formulation, and skin penetration dissolution tests were executed. The loading and delivery of OVA/CpG were quantified. Finally, immune responses from OVA/CpG encapsulated NPs in dMNA and in aqueous formulation were compared.

## Materials and Methods

### Materials

NE300 syringe pumps were purchased from ProSense B.V. (Oosterhout, The Netherlands). Pierce Micro bicinchoninic acid (BCA) protein assay kit, Qubit™ ssDNA Assay Kit, 500 µL Hamilton gastight, and polyether ether ketone (PEEK) capillary tubing were bought from Fisher Emergo B.V. (Landsmeer, the Netherlands). 10 mL Hamilton gastight syringes were purchased from Brunschwig Chemie B.V (Amsterdam, the Netherlands). A Teflon tube was purchased from Waters Chromatography B.V. (Etten-Leur, the Netherlands). One-piece fittings, female fitting Luer-lock adapters, two-piece adapters, interconnect tees were purchased from Mengel Engineering (Virum, Denmark). Silica capillary tubings were purchased from BGB Analytic Benelux B.V. (Harderwijk, the Netherlands). PLGA (acid terminated, lactide:glycolide 50:50, Mw 24 k -38 k), sodium dodecyl sulfate, sodium phosphate dibasic dihydrate, sodium phosphate monobasic dihydrate, pure sodium hydroxide pellets were purchased from Merck Chemicals B.V. (Amsterdam, the Netherlands). PVA (Mw 9 k), trehalose (Mw 378), PVP (Mw 40 k), and trypan blue were purchased from Millipore Sigma (Zwijndrecht, the Netherlands). Analytical grade dimethyl sulfoxide (DMSO) and acetonitrile purchased from Biossolve B.V. (Valkenswaard, the Netherlands). EndoFit™ Ovalbumin and CpG ODN 1826 (Class B) were purchased from InvivoGen, Bio-Connect B.V. (Huissen, the Netherlands). Spectra-Por® Float-A-Lyzer® G2 1 mL (1000 kDa MWCO) purchased from VWR International B.V. (Amsterdam, the Netherlands). Millex®-VV filter units (0.1 µm) was purchased from Merck Life Science N.V. (Amsterdam, the Netherlands). Silicon microneedle arrays were provided by Tyndall National Institute (Cork, Irland). SYLGARD 184 base silicone elastomer and curing agent silicone elastomer were purchased from Dow Corning (Midland, MI, USA). Epoxy glue was purchased from by Bison International B.V. (Goes, The Netherlands).

### Setup of the Microfluidic System

**Fig. 1:**
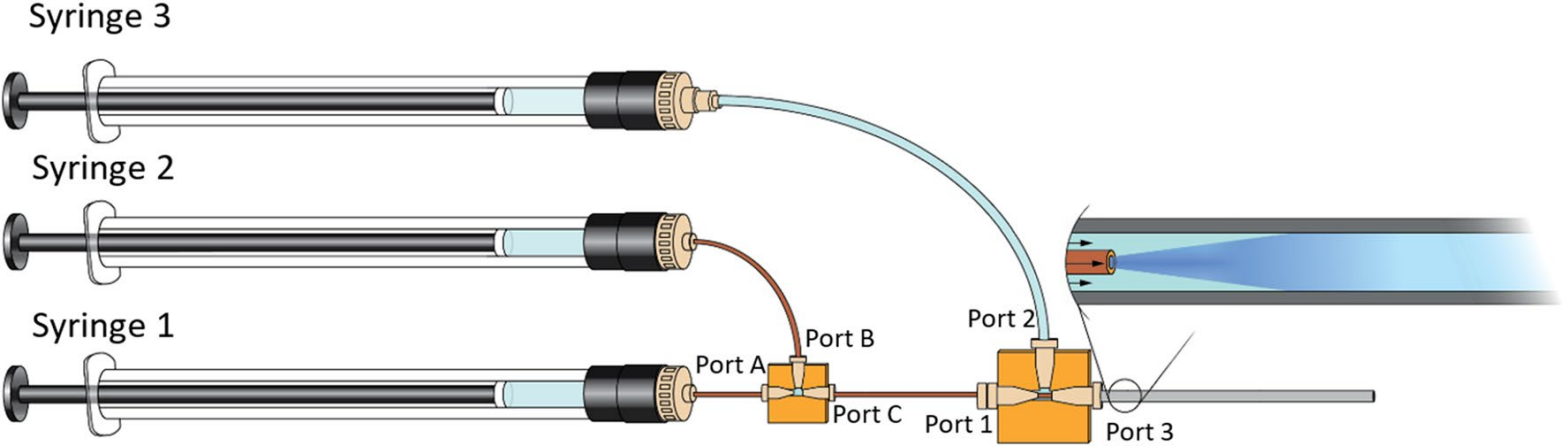
Schematic representation of the microfluidic-system setup. The fluid from Syringe 1 meets the fluid from Syringe 2 in a T-junction and the combined fluid meets the fluid in Syringe 3 in a co-flow where the combined fluid from Syringe 1 and 2 constitutes the inner fluid and the fluid from Syringe 3 constitutes the outer fluid.

The PLGA NPs were prepared with a three-syringe microfluidic system. The setup is depicted in Fig. 1. To assemble the microfluidic system, Luer-lock adaptors were screwed on Syringe 1, 2, and 3. Two 14-cm-long capillaries with inner diameters of 75 µm and 250 µm were attached to the Luer-lock adaptors with 360-µm fittings on Syringe 1 and Syringe 2, respectively. A 360-µm-interconnect tee with three ports in a T shape designated Port A, Port B, and Port C, where port A and C were opposite of each other and port B was positioned at an angle of 90° from Port A and B, was connected through Port A to the capillary from Syringe 1 with a 360-µm fitting. The capillary from Syringe 2 was connected to Port B with a 360-µm fitting. Port C was connected to a 14-cm-long capillary with an inner diameter of 250 µm with a 360-µm fitting. When the capillaries were attached, the capillaries were pushed to the end of the 360-µm fitting tip before insertion. A 1.6-mm-interconnect tee with three ports in a T shape designated Port 1, Port 2, and Port 3, where port 1 and 3 were opposite of each other and port 2 was positioned at an angle of 90°, was connected through Port 3 to a 7-cm-long piece of PEEK tube with a 1.6 mm fitting. A 360-µm-to-1.6-mm adapter was attached to the Luer-lock adapter on Syringe 3 and a 20-cm-long Teflon tube with an outer diameter of 1.6 mm was attached to it with a 1.6-mm fitting. The other end of the Teflon tube was attached to Port 2 with a 1.6-mm fitting. When the tubes were attached, they were first pushed fully into the 16-mm-interconnect tee before the 1.6-mm fitting was screwed on. A 1.6-mm-to-360-µm adapter was attached to Port 1. The capillary attached to Port C was inserted through a 360-µm fitting and pushed through the 1.6-mm-interconnect tee and PEEK tube, till it reached 1 cm through the PEEK tube, after which the 360-µm fitting was screwed on the 1.6-mm-to-360-µm adapter. To complete the setup, the syringes were mounted on the syringe pumps. When the formulations were collected from the end of the PEEK tube, the tube was held perpendicular to the sample collectors, and an initial volume of approximately 150 µL was discarded before the sample was tapped.

### Preparation of PLGA NPs with OVA and CpG

Two PLGA-NP formulations with OVA and CpG were prepared with a three-syringe microfluidic system: one to inject via a classical injection needle (aqueous-PLGA-NP formulation) and one used to prepare the dMNAs (dMNA-PLGA-NP formulation). For the preparation of the dMNA-PLGA-NP formulation, Syringe 1 of the microfluidic system was loaded with OVA and CpG dissolved in ultrapure water at concentrations of 4.0 mg/mL and 2.0 mg/mL, respectively. Syringes 2 and 3 were loaded with PLGA dissolved in acetonitrile at a concentration of 5.0 mg/mL and PVA (Mw of 9.5 kDa) dissolved in ultrapure water at a concentration of 14.1 mg/mL, respectively. The flow rates of the liquids dispensed from Syringes 1, 2, and 3 were set to 62.5, 625, and 2000 µL/min, respectively. After the syringe pumps were started the formulation was collected. A flow of nitrogen was used to evaporate the organic solvents from the formulation. Ultrapure water and 0.1 µm sterile filtered 100 mM phosphate buffer (75 mM Na2HPO4, 25 mM NaH2PO4) were added to achieve the final dMNA-PLGA-NP formulation, which consisted of 200 µg/mL OVA, 100 µg/mL CpG, 2.5 mg/mL PLGA, and 22.5 mg/mL PVA in 10 mM phosphate buffer (7.5 mM Na2HPO4, 2.5 mM NaH2PO4, pH 7.4).

We aimed to administer the same doses of OVA and CpG with the dMNAs and the injections with classical hypodermic needles in the immunization study. Therefore, the theoretically delivered quantities of these constituents from the dMNAs were calculated. One dose was delivered via two dMNAs, which theoretically would deliver 4.4 µg of OVA and 3.9 µg of CpG in total. To administer the same dose of OVA and CpG with the aqueous-PLGA-NP formulation (30 µL), the concentration of OVA in Syringe 1 was adjusted to 2.3 mg/mL. The remaining procedure was the same as for preparing the dMNA-PLGA-NP formulation. The final aqueous-PLGA-NP formulation consisted of 146 µg/mL OVA, 130 µg/mL CpG, 3.3 mg/mL PLGA, and 29.3 mg/mL PVA in 10 mM phosphate buffer. The soluble OVA and CpG were not removed from the dMNA-PLGA-NP formulation as dialysis would remove some of the PVA, which is a constituent needed to maintain stable dMNAs. To keep the aqueous-PLGA-NP and the dMNA-PLGA-NP formulations similar, the soluble OVA and CpG were also not removed from the aqueous-PLGA-NP formulation. The formulations were stored at 4 °C until use.

### Determination of the Hydrodynamic Particle Size and the Zeta Potential

The PLGA-NP formulations were analysed on a Zetasizer Nano ZS (Malvern Panalytical B.V., Almelo, the Netherlands) to determine the intensity-weighted mean hydrodynamic particle diameters (sizes) and PDIs with dynamic light scattering (detection angle of 173°), and the zeta potentials with laser Doppler electrophoresis. Before the measurements, the formulations were diluted 1:19 v/v in 10 mM phosphate buffer (n = 3).

### Determination of the Encapsulation Efficiencies of OVA/CpG

To compare the aqueous-PLGA-NP formulation with the dMNA-PLGA-NP formulation, the encapsulation efficiencies of OVA/CpG in the PLGA NPs were determined by measuring the total concentrations of OVA/CpG before and after dialysis as dialysis removes the OVA/CpG in the continuous phase. A sample was taken before the dialysis and 1 mL of each formulation was added to Float-A-Lyzer® dialysis device. Each dialysis device was submerged in 300 mL of 10 mM phosphate buffer (PB) and the formulations were dialysed for 72 h at 4 °C.

To determine the concentrations of OVA in the samples before and after dialysis, the samples were mixed in a volume ratio of 1:1 with a mixture of 30 vol% DMSO, 0.1 M NaOH, and 10 mg/mL sodium dodecyl sulfate, to disrupt the PLGA NPs, and incubated at 37 °C for 2 h. The standard curve was prepared with OVA in 15 vol% DMSO, 0.05 M NaOH, and 5 mg/mL sodium dodecyl sulfate. Each sample was prepared in triplicates and plated on a clear flat-bottom 96-well plate. The amounts of OVA were quantified with the micro BCA assay. The absorbance was measured at 562 nm with a plate reader (Tecan Spark®, Männedorf, Switzerland).

The concentrations of CpG in the samples before and after dialysis were quantified with a Qubit™ ssDNA Assay Kit. The calibration curve was made with CpG dissolved in ultrapure water and the solutions for the calibration curve were treated the same way as the samples. Each sample was prepared in triplicate. A volume of 40 µL from each sample was added to Eppendorf tubes and dried at 37 °C overnight to remove the water. They were reconstituted in 40 µL DMSO to disrupt the particles, and the vials were incubated at 37 °C for 2 h. The contents in the vials were spun down with a Microfuge® 18 centrifuge (1000 g, 5 min, Beckman Coulter Nederland B.V., Woerden, the Netherlands) and 30 µL of each sample was mixed with 270 µL of work reagent. After 2 min of equilibration time, 95 µL of each sample was plated on a black flat-bottom 96-well plate and 100 µL acetonitrile was added to each well. After 5 min, the fluorescence intensities (λ_ex_ 495 nm/λ_em_ 530 nm) were measured on a plate reader. The encapsulation efficiencies (EE%s) of OVA/CpG were calculated using the following equation:

$$\mathrm E\mathrm E\%=\frac{C\left(Sample\;after\;dialysis\right)}{C(Sample\;before\;dialysis)}\cdot\;100\%$$

### Preparation of PDMS Mould

The silicone microneedle arrays that serves as a template consists of nine (3 × 3) microneedles [35]. Each microneedle has a height of 500 µm and a base diameter of 330 µm. Nine (3 × 3) microneedle arrays are attached to the pedestal of a polymethylmethacrylate (PDMS) grid. The combination of this grid and nine silicone microneedle arrays is the master structure. In order to create the PDMS mould, a mixture of sylgard 184 base silicone elastomer and curing agent (10:1 weight ratio) is poured into the master structure and cured overnight at 60 °C [36]. The next day, the cured PDMS mould was removed from the master structure.

### Fabrication of dMNA and Screening of the dMNA Formulation

Securing the stability of PLGA NPs in dMNA is crucial to maintain the functionality of antigens. In order to find the most suitable polymer formulation for PLGA NPs incorporated dMNA, three different candidates of polymer formulation were used for dMNA production: 5% (w/v) PVA, 5% (w/v) PVP, and 30% (w/v) trehalose. These three different polymer formulations were screened based on the size and PDI of PLGA NPs in dMNAs.

To this end, dMNAs were fabricated with three different formulations as previously described [37]. First, empty (without OVA/CpG) PLGA NPs were added into each dMNA formulation with 1:4 of PLGA:polymer weight ratio, and the mixture was homogenised. Then, 90 µL of the mixture was loaded into the PDMS mould and centrifuged for 3 h at 25 °C with 11400 g. The centrifuged mould was dried at 37 °C overnight. The next day, silicone and epoxy glue were applied to each array to build a backplate. After another drying at 37 °C overnight, dMNAs were carefully removed from the mould. The shape and sharpness of dMNA were analysed with a brightfield microscope (Stemi 2000-C, Carl Zeiss Microscopy GmbH, Gottingen, Germany).

During the fabrication process, the size and PDI of PLGA NPs were measured three times using a Zetasizer as described in *Determination of the hydrodynamic particle size and the zeta potential* section (n = 3). They were measured (i) before and (ii) after adding into the polymer formulation. Subsequently, they were measured (iii) after re-suspending dMNA in 300 µL of PB (10 mM, pH 7.4). Among three candidates, the formulation that displayed the best retainment of PLGA NPs size and PDI (target size: < 200 nm, target PDI: < 0.3) was selected for further studies.

### Skin Penetration and Dissolution Tests

Penetration ability is a necessary function of dMNA in order to deliver the incorporated content into the skin. With the selected formulation from previous Section (5% (w/v) PVA with 1:4 PLGA:PVA ratio, see *Fabrication of dMNA and screening of the dMNA formulation* section), PLGA NP loaded dMNA was fabricated and skin penetration test was performed [35].

Human abdominal skin was collected from a local hospital after cosmetic surgery, and stored at -80 °C after removing the fat. Before use, the skin was thawed for an hour at 37 °C and stretched on parafilm-covered styrofoam. Next, the skin was wiped with 70% (v/v) ethanol to clean. dMNA was attached to an applicator (UFAM v1.0, uPATCH B.V., Delft, The Netherlands) to pierce the skin with a reproducible velocity. The dMNA was applied onto the skin with 65 ± 1 cm/s of velocity (n = 3), and removed after one second. Then, 75 µL of 0.4% trypan blue was applied on the dMNA applied skin site for 45 min. After removal of the trypan blue solution, the stratum corneum was removed by performing tape striping until the skin appeared shiny. Next, the skin was visualised using a brightfield microscope and the penetration efficiency was calculated by dividing the number of penetrated microneedles by the number of total microneedles in one dMNA.

Fast dissolution of microneedles is important to shorten the application time and facilitate the use of dMNA for patients. For the fast delivery, we aimed for 70% (volume) dissolution within 30 min. A dMNA was applied onto the skin in the same manner as for the penetration study. However, dMNA stayed for 30 and 60 min (n = 3) in the skin instead of being removed after one second. After removal, dMNA was imaged using a brightfield microscope and the leftover microneedle volume was calculated.

From the dissolution test, empty PLGA NPs loaded dMNA fabricated with the selected formulation did not show sufficient volume reduction even after 60 min. Therefore, this formulation required optimisation to ensure fast dissolution.

To this end, the total concentration of dMNA formulation was decreased from 5% (w/v) to 2.5% (w/v), and the weight ratio of PLGA:PVA was changed from 1:4 to 1:9. With this optimised formulation, PLGA NPs loaded dMNA was fabricated. Then, skin penetration and dissolution tests were repeated, whereby the dissolution time in the skin was 15 and 30 min.

### Quantification of OVA in OVA Encapsulated PLGA Loaded dMNA

Centrifugation is an effective method to produce dMNA. However, fabrication via this method leads to drug distribution in both the microneedles and backplate. Since the drug in the backplate will not be delivered into the skin, it is important to first quantify the antigen entrapped in only the microneedles in order to deliver the target dose (4 µg) of OVA. For this, soluble OVA/CpG loaded dMNA and OVA/CpG encapsulated PLGA NPs loaded dMNA were prepared (n = 3). For control groups, empty PLGA NPs loaded dMNAs and empty PLGA NPs with soluble OVA loaded dMNAs were prepared (n = 3).

To quantify the OVA in microneedles, nine microneedles were separated from the backplate (Figure S1) and reconstituted in 170 µL of DMSO/sodium dodecyl sulfate/NaOH solvent. After homogenising overnight, a BCA assay was performed. Briefly, 150 µL of homogenised solution was loaded into a 96-well plate followed by 150 µL of working reagent. The plate was incubated for 2 h at 37 °C, and the absorbance was measured at 562 nm by using a plate reader.

### Fabrication of Soluble OVA Loaded dMNA

In order to determine the effect of PLGA NPs on immune responses, both (i) soluble OVA/CpG loaded dMNA and (ii) OVA/CpG encapsulated PLGA NPs loaded dMNA were prepared for an immunisation study. To have the same dose of antigen in both groups, the same amount of OVA should be added to both of them during production. Therefore, the OVA amount in nine microneedles of OVA/CpG encapsulated PLGA NPs loaded dMNA was analysed in the previous section. Based on this quantification, the amount of OVA that should be added for soluble OVA/CpG loaded dMNA was determined which can have the same amount as OVA/CpG encapsulated PLGA NPs loaded dMNA.

For this, soluble OVA/CpG loaded dMNAs were fabricated with four different concentrations of OVA/CpG in 2.25% (w/v) PVA: 0.02% (w/v) OVA/0.01 (w/v) CpG%, 0.1% (w/v) OVA/0.05% (w/v) CpG, 0.2% (w/v) OVA/0.1% (w/v) CpG, and 0.5% (w/v) OVA/0.25% (w/v) CpG. As described in the previous section, nine microneedles were separated from the backplate. Then, OVA loading in nine microneedles was quantified using a BCA assay. Based on the quantified OVA in microneedles of four individual arrays (Figure S3), the required OVA amount for the production of soluble OVA/CpG loaded dMNA was determined.

### Quantification of CpG

Together with OVA, CpG was also encapsulated in PLGA NPs and added in dMNA in quantities of 50% w/w of the amount of OVA. To investigate the amount of CpG in microneedles, 0.05% (w/v) OVA and 0.025% (w/v) CpG were loaded in soluble CpG/OVA loaded dMNA based on the result of the studies described in the previous section. Empty PLGA NPs loaded dMNA and empty PLGA NPs with soluble OVA/CpG loaded dMNA were prepared for control groups.

Similar to OVA quantification described in *Quantification of OVA in OVA encapsulated PLGA loaded dMNAs* section, nine microneedles were separated and reconstituted in 170 µL of DMSO/SDS/NaOH. For the quantification of CpG, a Qubit™ ssDNA assay was executed as described in *Determination of the encapsulation efficiencies of OVA/CpG* section. For the calibration curve, 0.2 mg/mL and 0.15 mg/mL of CpG in Limulus amebocyte lysate water were prepared followed by a two-fold dilution. The fluorescence intensity was measured at λ_ex_ 538 nm/λ_em_ 488 nm using a plate reader.

### Animals

For the immunisation study, we used 35 female C57BL/6 mice, one OT-I mouse, which is a transgenic mouse on a C57BL/6 genetic background with T-cell receptors that pair with CD8 and recognise OVA_257-264_ presented on MHC class I (haplotype H-2 Kb) molecules, and two OT-II mice, which are transgenic mice on a C57BL/6 genetic background with T-cell receptors that pair with CD4 and recognise OVA_323-339_ on MHC class II (haplotype I-A^b^) molecules. They were 7–12 weeks old at the start of the experiment and were kept under standard laboratory conditions at the animal facility of Leiden Academic Centre for Drug Research, Leiden University. The animal experiment was approved by the ethical committee of Leiden University, and the animal work was performed in compliance with the Dutch government guidelines and Directive 2010/63/EU of the European Parliament.

### Immunisation

To determine if the aqueous formulations and dMNA were able to activate OVA-specific T cells, an immunisation study was performed in mice according to the schedule (Table 1).

**Table 1:** The Schedule of Immunisation Study

Day 1	Transfer OVA-specific CD8^+^ T cells and OVA-specific CD4^+^ T cells
Day 2	Immunise mice
Day 9	Harvest blood and spleens

On day 1, each of the 35 C57BL/6 mice was injected in the tail vein with 50,000 OVA-specific CD8^+^ T cells which were isolated from a spleen collected from an OT-I mouse and 100,000 OVA-specific CD4^+^ T cells which were isolated from two spleens collected from two OT-II mice [38] using a BD Microlance™ 3 0.3 × 13 mm needle (Becton Dickinson N.V., Vianen, Holland).

On day 2, mice were weighed, marked, and allocated into seven groups of five mice, balanced with regard to weight and age. Each group was randomly assigned to seven different vaccine regimens (Table 2).

**Table 2:** Information on Mice Group for Immunisation Study. *Dose in dMNAs was Estimated Based on Dissolution

Mice group	Regimens	Administration site	Target dose OVA/CpG (µg)
1	Empty dMNA (negative control)	Flank	0/0
2	Soluble OVA/CpG loaded dMNA*	Flank	4.4–4.9/ 3.9–4.3
3	OVA/CpG encapsulated PLGA NPs loaded dMNA*	Flank	4.4–4.9/ 3.9–4.3
4	Soluble OVA/CpG loaded dMNA*	Ear	4.4–4.9/ 3.9–4.3
5	OVA/CpG encapsulated PLGA NPs loaded dMNA*	Ear	4.4–4.9/ 3.9–4.3
6	OVA/CpG in PBS	Flank	4.4/3.9
7	Aqueous-PLGA-NP	Flank	4.4/3.9

The mice group 1–3, 6, and 7 had their flanks shaved. The mice group 1–5 were anaesthetised by intraperitoneal injection with ketamine (100 mg/kg) and xylazine (10 mg/mL) for 10 min before administration of dMNAs (two dMNAs per mouse). The mice were placed on a Heatel Teera Heatmat (Heatel B.V., Poeldijk, the Netherlands) and ophthalmic ointment was applied to their eyes. The mice group 2–5 were expected to receive 4.4–4.9 µg OVA and 3.9–4.3 µg CpG. The mice enrolled in Regimen 1 did not receive OVA/CpG. The dMNA was administrated into the skin for 30 min one at a time on either the flank or the ear. The dMNA was visualised using a bright-field microscope after being removed from the skin.

The mice group 6 and 7 received 4.4 µg OVA and 3.9 µg CpG intradermally (flank) in a volume of 30 µL by using BD micro-fine™ + Demi U100 0,3 mL 30G insulin needles (Fisher Emergo B.V., Landsmeer, the Netherlands). Unfortunately, two mice died because of the anaesthesia (mice receiving Regimen 2 or 4), and one mouse receiving Regimen 2 was euthanised by cervical dislocation after reaching humane endpoints (limping and 17% weight reduction). On day 9, blood was collected by tail bleeding in micorvette® CB K_2_EDTA 300 µL tubes (Sarstedt B.V., Etten-Leur, the Netherlands). The mice were euthanised by cervical dislocation whereafter the spleens were harvested. The spleens were immersed in PBS and the blood and spleens were kept on ice till further use.

### Flow Cytometric Analysis of CD8^+^ and CD4^+^ T Cells

To assess the OVA-specific T-cell responses in the immunised mice, the cells in the blood and spleens were stained with fluorophore-tagged antibodies. The spleens were strained to obtain single-cell suspensions and then erythrocytes were depleted from the splenocyte-containing single-cell suspensions using ACK lysing buffer. Hereafter, the splenocytes were resuspended in 2 mL cRPMI and 100 µL of each suspension was added to a 96-well U-bottom plate. The erythrocytes in the blood were depleted in a similar way: the blood cells were suspended in 2 mL ACK lysing buffer and the lysis was stopped after 5 min by adding 5 mL cRPMI medium. The remaining blood cells were washed in 5 mL cRPMI medium and resuspended in 300 µL cRPMI medium. 100 µL of each blood-cell suspension was added to the 96-well round bottom plate. The plate with the blood and spleen cells was centrifuged (5 min, 550 g, 4 °C) and the supernatants were removed. The cells were resuspended in 100 µL surface marker staining solution (containing the fluorophore-tagged antibodies CD45.1 PE-Dazzle-594 (clone A20), Thy1.2 PE/Cyanine7 (clone 53–2.1), CD8a Brilliant Violet 510 (clone 53–6.7) (all from BioLegend Europe B.V., Amsterdam, the Netherlands), CD4 eFlour 450 (clone GK1.5), and 7-AAD Viability Staining Solution (live/dead marker) (both from eBioscience™, Fisher Emergo B.V.) in FACS buffer (1 mM EDTA, 2% fetal bovine serum, 0.1% sodium azide)), and the plate was covered with aluminium foil and incubated for 30 min at 4 °C. The plate was centrifuged (5 min, 550 g, 4 °C) and the supernatants were removed. The cells were washed two times and resuspended in 100 µL FACS buffer. Hereafter, the cells were analysed by flow cytometry (CytoFLEX S V4-B2-Y4-R3, Beckman Coulter, California, USA) with the acquisition software CytExpert (v2.3.1.22, Beckman Coulter). The sample flow rate and recorded volume were set to 60 µL/min and 80 µL, respectively. Data were analysed and manually compensated by using FlowJo software v10 (Treestar, Oregon, USA). The live CD8^+^ or CD4^+^ T cells were detected by first gating for single cells, then live (live/dead marker negative) T (Thy1.2 marker positive) cells, and subsequently CD4^+^ (CD4 marker positive) or CD8^+^ (CD8a marker positive) cells. The OVA-specific cells in the live CD4^+^ or CD8^+^ T cell populations were detected by measuring the frequency of CD45.1 (donor cells from the OT-I and OT-II mice) positive cells.

### Statistical Analysis

The data from the animal experiment was analysed in GraphPad Prism® version 8.0.1 (GraphPad Software, CA, USA). The statistical significance was determined with a one-way analysis of variance, followed by Bonferroni's multiple comparisons test and P < 0.05 was considered statistically significant (*P < 0.05, **P < 0.01, ***P < 0.001, ****P < 0.0001).

## Results

### Characterisation of PLGA NPs and Encapsulation Efficiencies of OVA/CpG

The aqueous-PLGA-NP formulation and the dMNA-PLGA-NP formulation used to fabricate dMNA were prepared with the microfluidic system. When preparing the two formulations, the setup of the microfluidic system remained unchanged including concentrations of CpG, PLGA, and PVA in the syringes and the flow rates. The altered components were the OVA concentration in Syringe 1 and the final concentration of the two formulations. The OVA concentration in Syringe 1 was lower for the aqueous-PLGA-NP formulation than the dMNA-PLGA-NP formulation. The CpG:OVA:PLGA:PVA weight ratio was 1:2:25:225 for the dMNA-PLGA-NP formulation and 1:1.13:25:225 for the aqueous-PLGA-NP formulation resulting in a higher OVA concentration in the aqueous-PLGA-NP formulation than in the dNMA-PLGA-NP formulation. In the final formulations, the concentration of CpG, PLGA, and PVA was slightly higher for the aqueous-PLGA-NP formulation as it was concentrated more. The two formulations were characterised with regard to size, PDI, zeta potential, and encapsulation efficiencies of OVA/CpG (Table 3). The minor change in the method did not affect the physicochemical characteristics of the PLGA NPs massively. Both formulations were monodisperse, indicated by having PDIs below 0.1, sizes around 100 nm and slightly negative zeta potentials. OVA/CpG were effectively encapsulated into the PLGA NPs in both formulations with encapsulation efficiencies above 35%. The encapsulation efficiencies of OVA/CpG were highest in the dMNA-PLGA-NP formulation. Soluble OVA/ CpG were not removed. Therefore, OVA/CpG are only partly encapsulated in the PLGA NPs when it is administered into the mice.

**Table 3:** Physicochemical Characteristics of the PLGA NPs with OVA/CpG used for the Aqueous-NP Formulation and the dMNA. The Physicochemical Characteristics of the dMNA-NP Formulation are Measured while the Formulation is Liquid i.e. Before they are Added to the dMNA

NP formulation	Size (nm)	PDI	ZP (mV)	EE%	
				OVA	CpG
Aqueous-PLGA-NP formulation	96.1 ± 0.4	0.090 ± 0.023	-0.85 ± 0.67	36.7	35.6
dMNA-PLGA-NP formulation	100.2 ± 1.7	0.097 ± 0.015	-1.94 ± 0.62	52.7	45.0

Average ± SD of three technical replicates. Size: intensity-weighted mean hydrodynamic particle diameters, PDI: polydispersity index, ZP: zeta potential, EE%: encapsulation efficiency

### Fabrication of dMNA and Screening of the dMNA Formulation

**Fig. 2:**
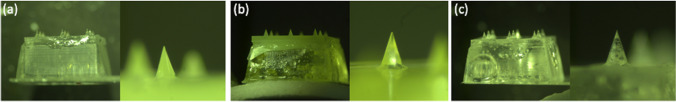
Fabricated dMNAs with (**a**) 5% (w/v) PVA, (**b**) 5% (w/v) PVP, and (**c**) 30% (w/v) trehalose.

**Fig. 3:**
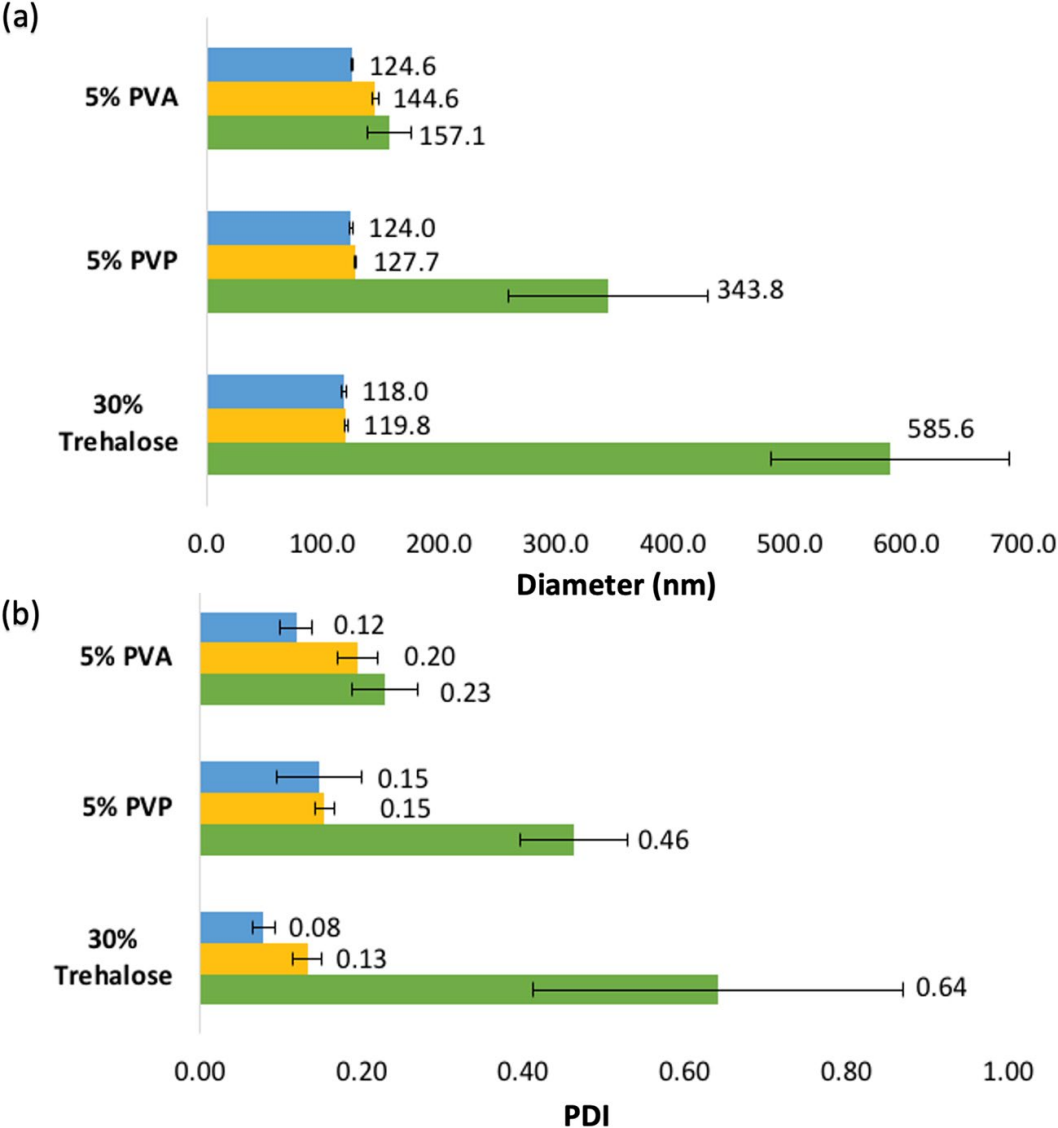
The (**a**) average size and (**b**) PDI of PLGA NPs in three different formulations and re-suspended dMNAs. (blue: before adding PLGA NPs to the dMNA formulation, yellow: after adding PLGA NPs to the dMNA formulation, green: after re-suspending the dMNA).

dMNAs were fabricated with three different polymer formulation: 5% (w/v) PVA, 5% (w/v) PVP, and 30% (w/v) trehalose. All three formulations successfully formed nine sharp microneedle tips in each array (Fig. 2). Therefore, PLGA NPs could be incorporated in dMNAs with all three polymer formulations. The size and PDI of PLGA NPs were measured (i) before and (ii) after adding them to the formulation and also (iii) after re-suspending dMNA. As shown in Fig. 3a, the mean size of PLGA NPs was slightly increased after adding them to the formulations compared to before adding them. It increased 16.1% for 5% (w/v) PVA, 3.0% for 5% (w/v) PVP, and 1.5% for 30% (w/v) trehalose. These values considerably increased after re-suspending PVP dMNA (177.3%) and trehalose dMNA (396.3%). Only PVA dMNA displayed a slight increase in size (26.1%). PDI also showed similar trends. The size and PDI of PLGA NPs in only PVA dMNA were within the target range which were below 200 nm and 0.3, respectively (Fig. 3b). Therefore, 5% (w/v) PVA was selected as the dMNA formulation for further studies.

### Skin Penetration and Dissolution of dMNA

**Fig. 4:**
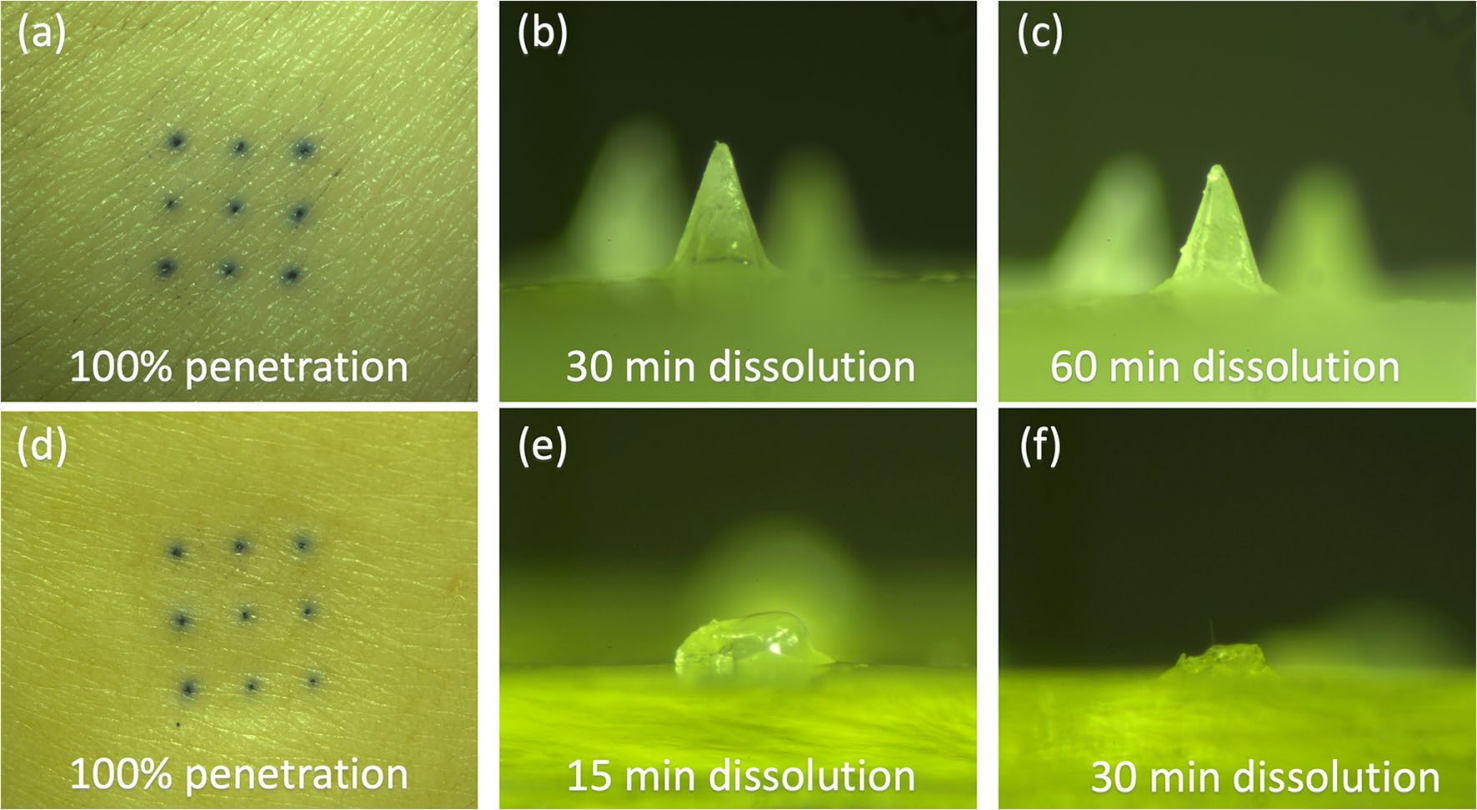
(**a**) Penetrated skin and (**b**) dissolved microneedle after 30 min (**c**) 60 min of dissolution with 5% (w/v) PVA dMNA with 1:4 PLGA:PVA ratio. (**d**) Penetrated skin and (**e**) dissolved microneedle after 15 min (**f**) 30 min of dissolution with 2.5% (w/v) PVA dMNA with 1:9 PLGA:PVA ratio.

Based on the results of PLGA NPs stability in the previous section, 5% (w/v) PVA was selected as a dMNA formulation. In order to investigate the mechanical strength and dissolution ability of dMNA, skin penetration and dissolution tests were performed. The penetration study demonstrated excellent penetration efficiency of 100% as all nine microneedles penetrated the skin (n = 3, Fig. 4a). In the dissolution test, however, only 3.6 ± 0.4% and 5.7 ± 0.4% of microneedle volume were dissolved in the skin within 30 and 60 min, respectively (Fig. 4b-c).

Hence, the dMNA formulation was further optimised by reducing the total concentration of dMNA formulation (2.5% (w/v)) and decreasing the proportion of PLGA NPs in the formulation (1:9 PLGA:PVA weight ratio). Skin penetration and dissolution tests were executed again with dMNAs produced with the optimised formulation. As a results, 2.5% (w/v) PVA dMNA (1:9 PLGA:PVA) demonstrated smilar penetration efficiency (100%) and a faster dissolution ability compared to 5% (w/v) total concentration of dMNA formulation with 1:4 PLGA:PVA weight ratio (Fig. 4d). Within 15 and 30 min, 55.8 ± 0.3% and 73.2 ± 2.8% of the microneedle volume dissolved, respectively (Fig. 4e-f). For further studies, 2.5% (w/v) total concentration of dMNA formulation with 1:9 PLGA:PVA weight ratio was used.

### Quantification of OVA

dMNA fabrication using a centrifugation method results in a substantial amount of antigen loading in the backplate. Therefore, quantifying OVA in only microneedles is necessary to determine the delivered dose. Theoretically, 225 µg of PLGA NPs and 18 µg of OVA were expected to be loaded in each dMNA (including backplate) since the ratio of OVA:PLGA:PVA was 2:25:225. Based on the BCA assays, it was determined that 3.2 ± 0.4 µg of OVA was loaded in nine microneedles (17.8% of the total amount loaded in one dMNA). Based on the 70–76% dissolved volume of the microneedles (see the previous section), 2.2–2.4 µg of OVA was estimated to be delivered to the skin.

### Fabrication of Soluble OVA Loaded dMNA

For the immunisation study, soluble OVA/CpG loaded dMNA and OVA/CpG encapsulated PLGA NPs loaded dMNA should carry an identical dose in nine microneedles which is 3.2 µg (see the previous section). Therefore, four different concentrations of OVA/CpG loaded dMNAs were prepared, and OVA in nine microneedles was quantified. As a result, 1.6 µg, 5.6 µg, 10.4 µg, and 16.1 µg of OVA were loaded in nine microneedles of 0.02% (w/v), 0.1% (w/v), 0.2% (w/v), and 0.5% (w/v) of OVA loaded dMNAs, respectively. Based on these results, a calibration curve was generated (Figure S3) to display the OVA concentration of four different dMNAs and the corresponding OVA amount in nine microneedles. From the calibration curve, the OVA concentration for soluble OVA/CpG loaded dMNA production was determined which can carry 3.2 µg OVA in nine microneedles. Hence, 0.05% (w/v) of OVA should be loaded in soluble OVA/CpG loaded dMNA in order to carry the same OVA amount as OVA/CpG encapsulated PLGA NPs loaded dMNA.

### Quantification of CpG

In PLGA NPs, CpG was also encapsulated as an adjuvant with a half weight ratio of OVA. Therefore, 0.05% (w/v) of OVA and 0.025% (w/v) of CpG were added in soluble OVA/CpG loaded dMNA based on the result of the previous section. By using the Qubit™ ssDNA assay, it was determined that 2.81 ± 0.04 µg of CpG was carried in nine microneedles. After 70–76% of dissolution, 2.0–2.1 µg of CpG is expected to be delivered to the skin.

###  Immunisation study

An immunisation study was performed to determine whether the presence of PLGA NPs and the administration form and location affects the T-cell responses *in vivo*. Transferred OVA-specific T cell mice received seven different regimens which consisted of dMNAs or aqueous formulations with either soluble OVA/CpG or OVA/CpG partly encapsulated in PLGA NP (Table 3). The dMNAs were administered at two different locations: the flanks or the ear pinnae. Seven days after the immunisation, the T-cell responses in the blood and spleen were analysed by flow cytometry. The gating strategies are shown in Figure S2.

**Fig. 5:**
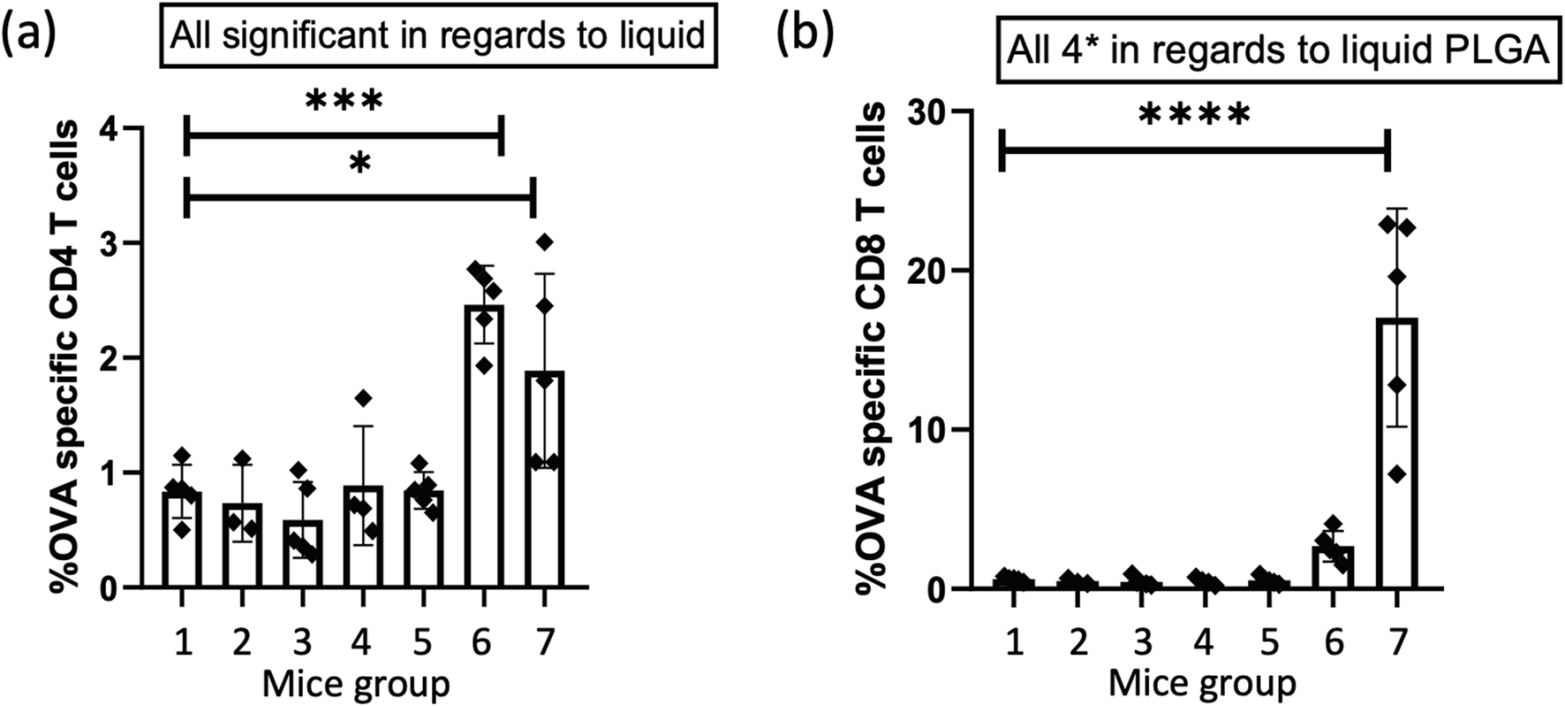
The percentage of OVA-specific CD4^+^ (**a**) and CD8^+^ (**b**) T cells of the total amount of CD4^+^ and CD8^+^ T cells, respectively, in the harvested blood. (mean ± SD, n = 5, except for flank MNA CpG and OVA n = 3, and ear MNA CpG and OVA n = 4). The formulations were compared with the empty dMNA, which was the negative control, for statistical significance. *P < 0.05, **P < 0.01,***P < 0.001, ***P < 0.0001.

**Fig. 6:**
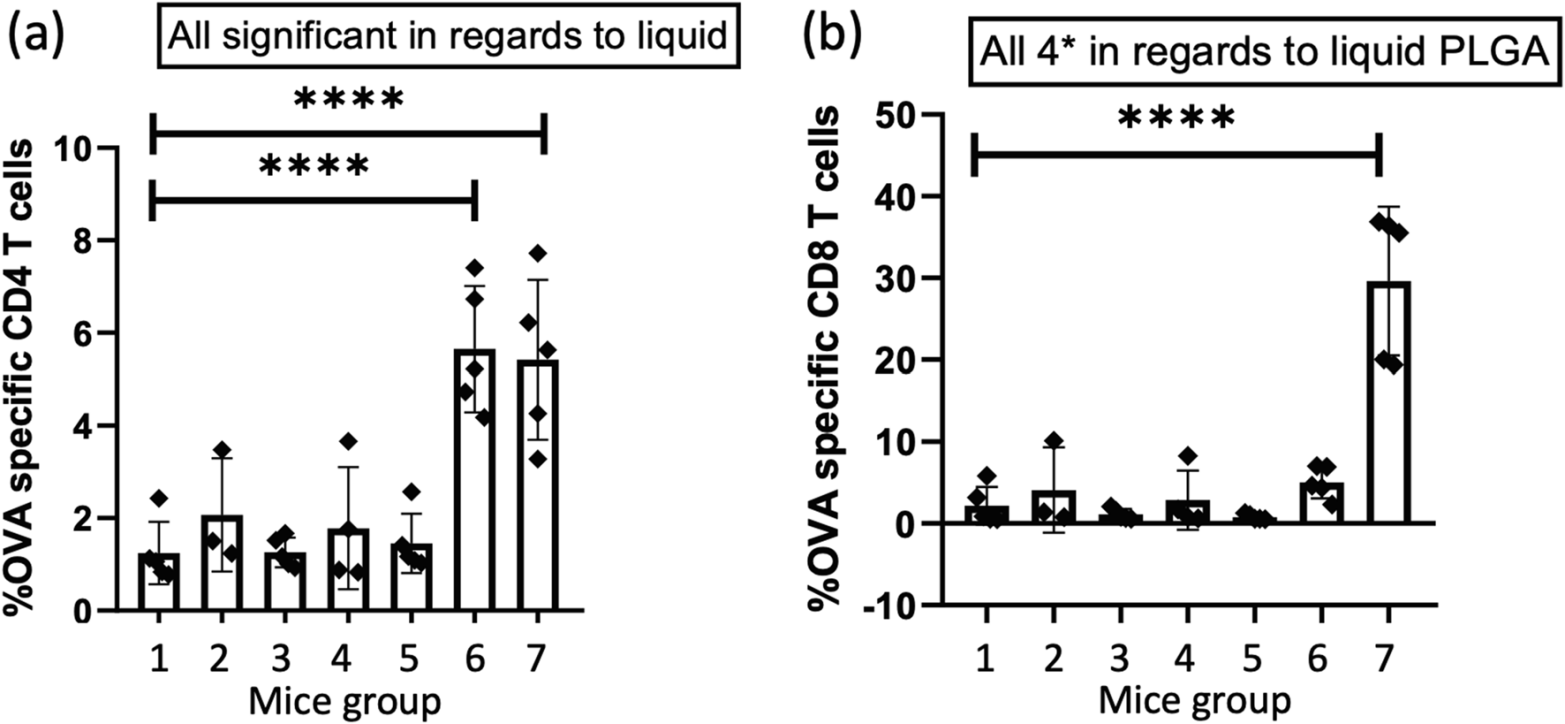
The percentage of OVA-specific CD4^+^ (**a**) and CD8^+^ (**b**) T cells of the total amount of CD4^+^ and CD8^+^ T cells, respectively, in the harvested spleens. (mean ± SD, n = 5, except for flank MNA CpG and OVA n = 3, and ear MNA CpG and OVA n = 4). The formulations were compared with the empty dMNA, which was the negative control, for statistical significance. *P < 0.05, **P < 0.01,***P < 0.001, ***P < 0.0001.

The two aqueous formulations: OVA/CpG in PBS (group 6) and the aqueous PLGA NPs formulation (group 7) induced high expansion of the transferred OVA-specific CD4^+^ T cells in the blood and spleen cells. OVA/CpG in PBS induced the highest response of OVA-specific CD4^+^ T cells in the blood (2.5 ± 0.3% of the CD4^+^ T-cell population) (Fig. 5a) and it was significantly higher than the response from the negative control (group 1, P < 0.001). The aqueous PLGA NPs formulation also induced a significantly higher OVA-specific CD4 + T-cell response (1.9 ± 0.8% of the CD4^+^ T-cell population) (P < 0.05) compared to the negative control. There was no significant difference between the CD4 + T-cell responses induced by the two aqueous formulations. The same pattern was seen for the OVA-specific CD4^+^ T-cell responses in the spleens (Fig. 6a), however, the responses were higher. OVA/CpG in PBS induced the highest response of OVA-specific CD4^+^ T cells in the spleen (5.7 ± 1.4% of the CD4^+^ T-cell population), and it was significantly higher than the negative control (P < 0.0001). The aqueous PLGA NPs formulation also induced a significantly higher OVA-specific CD4^+^ T-cell response (5.4 ± 1.7% of the CD4^+^ T-cell population) (P < 0.0001) compared to the negative control. Similar to the blood cells, there was no significant difference between the CD4^+^ T-cell responses induced by the two aqueous formulations (group 6 and 7). The aqueous PLGA NPs formulation induced the highest OVA-specific CD8^+^ T-cell response in the blood (17.0 ± 6.9% of the CD8^+^ T-cell population) (Fig. 5b), this was statistically higher compared to the negative control (P < 0.0001). OVA/CpG in PBS induced an OVA-specific CD8 + T-cell response of 2.7 ± 1.0%, however, this was not statistically significant compared to the negative control, though it was higher than the 0.4–0.6% baseline established by the dMNA with (group 3 and 5) and without PLGA NPs (group 2 and 4). In this case, the aqueous PLGA NPs formulation induced a significantly higher OVA-specific CD8^+^ T-cell response (P < 0.0001) compared to OVA/CpG in PBS. The same pattern was seen in the spleen, however, the responses were higher. The aqueous PLGA NPs formulation induced an OVA-specific CD8^+^ T-cell response of 29.6 ± 9.1% (of the CD8^+^ T-cell population) (Fig. 6b), which was statistically significant compared to the negative control and the response in the spleen was again higher than the CD8^+^ T-cell response in the blood.

The regimens with dMNAs (group 2–5) did not significantly change the OVA-specific CD4^+^ T-cell responses compared to the negative control in the blood cells, and there was no significant change in the responses between the dMNAs inserted into the flank (group 2 and 3) and the ear (group 4 and 5). Neither was there a significant change in the responses between the dMNAs with (group 3 and 5) and without PLGA NPs (group 2 and 4), where all of the dMNAs induced OVA-specific CD4^+^ T-cell responses of 0.6–0.9% in the CD4^+^ T-cell populations. The same pattern was observed for the OVA-specific CD4^+^ T-cell responses in the spleens, however, the T-cell responses were slightly higher for all of the regimens (Fig. 6a), where the dMNAs all induced around 1.3–1.5% of the CD4^+^ T-cell population. The dMNA formulations (group 2–5) did not induce significant CD8^+^ T-cell responses compared to the control in the blood and spleen either.

## Discussion

In this study, we developed OVA/CpG encapsulated PLGA NPs loaded dMNA. NPs work as adjuvant and enhance taking up of antigens by APCs. By delivering them to the skin which has abundant APCs, it is expected to boost the induction of T cells. For this, we chose two different approaches: (i) encapsulate antigens and adjuvant in PLGA NPs and (ii) deliver them intradermally using dMNA.

We chose to incorporate the antigen (OVA) and the molecular adjuvant (CpG) into the formulations. OVA is readily available and is often used to study antigen-specific immune responses in mice [39]. By performing an adoptive transfer of T cells from OT mice, whose T cells recognize specific OVA-derived peptide residues, the T-cell response is enlarged compared to wild-type mice, which normally require multiple vaccinations before OVA-specific immune responses can be measured. We chose CpG as adjuvant as previous studies have shown that subunit vaccines against SARS-CoV and HIV-1 containing CpG have activated dendritic cells [40] and induced specific Th1 and CD8^+^ T-cell responses [26, 27]. We incorporated OVA/CpG into the PLGA NPs with a modular microfluidic system. The NPs sizes for the immunisation study were aimed at 100 nm as particles under 200 nm are more easily taken up by DC and supposedly give Th1 and CD8^+^ T-cell responses [18, 41]. Furthermore, a previous study have compared PLGA NPs with OVA/CpG with sizes from 300 nm to 17 µm. The PLGA particles with 300 nm induced the highest expression of the DC activation markers CD86 and MHC class I, compared to naïve cells, soluble OVA/CpG and PLGA microparticles with sizes of 17 µm, 7 µm, 1 µm. We succeeded at producing two formulations with monodisperse particles at around 100 nm with the microfluidic system. The sizes are normally larger with conventional methods, as double-emulsion and nanoprecipitation methods lead to PLGA NP with a minimum sizes at approximately 150 nm [42, 43]. Both of the NP formulations had zeta potentials of around -1 mV. This is a bit higher than PLGA particles prepared without PVA (-32 mV) [28]. This is likely due a PVA layer on the surface of the NPs that shields the charge [44]. The soluble OVA/CpG was not removed from the PLGA-NP formulations because PVA, which is a small molecule (Mw 9 kDa), also would be removed during the dialysis. PVA is both a surfactant and the material chosen to produce the dMNA. Its concentration is crucial for dMNA production since mechanical strength of dMNA is proportional to the PVA concentration. We measured the encapsulation efficiencies of OVA/CpG. OVA had an encapsulation efficiency of 37–57%, which is higher than generally obtained with other microfluidic devices [28], but lower than via the conventional double emulsion method [42]. However, the methods are not directly comparable, as some of the small PLGA particles with a lower encapsulation efficiency probably is removed during the conventional method.

We also chose dMNA to deliver vaccines to the skin which has high population of APCs. The fabrication of PLGA NPs incorporated dMNA was successful. In terms of size and PDI, the NPs maintained stability even after being loaded into dMNA. Also, PLGA NPs loaded dMNA proved adequate mechanical strength by penetrating the skin effectively. The immunogenicity was expected to be elevated by introducing NPs in dMNA, since NPs allows targeted co-delivery of antigen and adjuvant. Unexpectedly, the immune responses from mice groups that received both soluble OVA/CpG and OVA/CpG encapsulated PLGA NPs using dMNAs (group 2–5) were significantly lower compared to those who received aqueous formulations (group 6 and 7). In recent studies, the combination of NPs and dMNAs failed to elicit immune responses. For example, OVA and poly(I:C) encapsulated PLGA NPs loaded dMNA failed to evoke CD8^+^ T-cell responses [42].

In our study, the major reason for the poor immune responses from dMNA-received groups was poor dissolution and, as a result, insufficient dosages. Through an *in vitro* skin dissolution test, it was proved that the majority of the microneedle volume was dissolved in human abdominal skin within 30 min (Fig. 4F). However, dMNA applied on *in vivo* mouse skin showed poor dissolution with the same application time (30 min) compared to the *in vitro* human skin so that the leftover volume of microneedle was larger than *in vitro* dissolution test (Figure S4). This poorer dissolution in mouse skin might be resulted due to uneven surface of mouse skin compared to human skin, since the human skin was stretched on the flat and smooth Styrofoam during the *in vitro* skin dissolution test.

Poor dissolution also could be caused by uneven distribution of PLGA NPs in microneedle. Centrifugal force made PLGA NPs concentrated in the tip of microneedles due to their weight, as observed by using fluorescently-labelled PLGA NPs loaded microneedles (Figure S5). Since microneedle dissolution starts from the tip, the localisation of hydrophobic PLGA NPs in the tip disturbs rapid dissolution. Therefore, it is important to select proper production methods for the dMNA, which can distribute the drug formulation homogeneously in the microneedle. For example, filling the PDMS mould by spraying drug formulations can make homogeneously distributed microneedles [45]. Moreover, this spraying technique also can load multiple layers of drug and controlling the release by using multiple sprays. However, sprayed formulation is spread over the mould including the surface out of microneedles so that brings antigen waste. Elongating the dispensed drug formulation by placing it between two plates also contributes to the even distribution of drug formulation in the microneedle [46]. With this droplet-born air blowing technique, the length of microneedle is controllable and antigen waste can be avoided. Dispensing drug formulations is another way to reduce the localisation of NPs in the tip [35]. It can save antigen by loading the formulation only into the tips and automised system is possible by adding moving stages. However, the dispensable formulation is limited to the low viscosity.

## Conclusion

To induce high antigen-specific T-cells responses, we chose two approaches: (i) incorporate the antigen and the molecular adjuvant into particles as they are more readily taken up by DCs which are key players in the induction of antigen-specific T-cell responses and (ii) deliver the formulations to a DC rich organ using dMNA. To achieve this, we developed OVA/CpG encapsulated PLGA NPs, and incorporated them into dMNA.

The fabrication of dMNA was successful as it displayed complete penetration efficiency with a major dissolution. Upon intradermal injection of aqueous formulation the PLGA NPs induced high CD4^+^ T-cell and superior CD8^+^ T-cell responses in the blood and spleen, showing the powerful approach of formulating for improved APCs uptake. 

### Supplementary Information

Below is the link to the electronic supplementary material.

Supplementary file1 (DOCX 10604 KB)

## Data Availability

All data supporting the findings of this study are included in this paper.

## Abbreviations

APCs: Antigen presenting cells
ACK: Ammonium-chloride-kalium (potassium)
BCA: Bicinchoninic acid
CpG: CpG ligonucleotide
dMNA: Dissolving microneedle array
DMSO: Dimethyl sulfoxide
EE%: Encapsulation efficiency
NPs: Nanoparticles
OVA: Ovalbumin
PLGA: Poly(D,L-lactic-*co*-glycolic acid)
PDI: Polydispersity index
PVA: Polyvinyl alcohol
PVP: Polyvinylpyrrolidone
TLR: Toll-like receptor

## References

[1] Hofmann F, Kralj N, Beie M. [Needle stick injuries in health care - frequency, causes und preventive strategies]. Gesundheitswesen [Internet]. 2002 May 1 [cited 2023 Mar 22];64(5):259–66. Available from: https://europepmc.org/article/med/12007067. 10.1055/s-2002-28353 12007067

[2] Prüss-Üstün A, Rapiti E, Hutin Y. Estimation of the global burden of disease attributable to contaminated sharps injuries among health-care workers. Am J Ind Med [Internet]. 2005 Dec 1 [cited 2023 Mar 22];48(6):482–90. Available from: https://onlinelibrary.wiley.com/doi/full/10.1002/ajim.20230. 10.1002/ajim.20230 16299710

[3] Biswas J, Dhali A, Panja S, Karpha K, Nath S, Dhali GK. Effect of hypodermic needle versus safety lancet on the fear and anxiety of needle prick among undergraduate medical students during hematology practical: a cohort study from a resource-limited setting. Cureus [Internet]. 2022 Jul 29 [cited 2023 Mar 22];14(7). Available from: /pmc/articles/PMC9420539/. 10.7759/cureus.27458 PMC9420539 36060377

[4] Ita K (2017). Dissolving microneedles for transdermal drug delivery: Advances and challenges. Biomed Pharmacother.

[5] Liu S, Jin MN, Quan YS, Kamiyama F, Kusamori K, Katsumi H (2014). Transdermal delivery of relatively high molecular weight drugs using novel self-dissolving microneedle arrays fabricated from hyaluronic acid and their characteristics and safety after application to the skin. Eur J Pharm Biopharm.

[6] Zhang L, Guo R, Wang S, Yang X, Ling G, Zhang P (2021). Fabrication, evaluation and applications of dissolving microneedles. Int J Pharm.

[7] Chen S, Matsumoto H, Moro-oka Y, Tanaka M, Miyahara Y, Suganami T, *et al*. Microneedle-array patch fabricated with enzyme-free polymeric components capable of on-demand insulin delivery. Adv Funct Mater [Internet]. 2019 Feb 1 [cited 2023 Apr 24];29(7):1807369. Available from: https://onlinelibrary.wiley.com/doi/full/10.1002/adfm.201807369.

[8] Adams SB, Acvs D, Moore GE, Acvim D, Elrashidy M, Mohamed A, *et al.* Effect of Needle Size and Type, Reuse of Needles, Insertion Speed, and Removal of Hair on Contamination of Joints with Tissue Debris and Hair after Arthrocentesis. Veterinary Surgery [Internet]. 2010 Aug 1 [cited 2023 Apr 24];39(6):667–73. Available from: https://onlinelibrary.wiley.com/doi/full/10.1111/j.1532-950X.2010.00649.x. 10.1111/j.1532-950X.2010.00649.x 20345539

[9] Mungmunpuntipantip R, Wiwanitkit V. Cost-utility-safety analysis of alternative intradermal versus classical intramuscular COVID-19 vaccination. Int J Physiol Pathophysiol Pharmacol [Internet]. 2022 [cited 2023 May 8];14(2):129. Available from: /pmc/articles/PMC9123469/. PMC9123469 35619662

[10] Egunsola O, Clement F, Taplin J, Mastikhina L, Li JW, Lorenzetti DL, *et al*. Immunogenicity and safety of reduced-dose intradermal vs intramuscular influenza vaccines: a systematic review and meta-analysis. JAMA Netw Open [Internet]. 2021 Feb 1 [cited 2023 Feb 26];4(2):e2035693–e2035693. Available from: https://jamanetwork.com/journals/jamanetworkopen/fullarticle/2776045. 10.1001/jamanetworkopen.2020.35693 PMC7873776 33560425

[11] Di Pasquale A, Preiss S, Da Silva FT, Garçon N (2015). Vaccine adjuvants: From 1920 to 2015 and beyond. Vaccines.

[12] Benne N, van Duijn J, Kuiper J, Jiskoot W, Slütter B (2016). Orchestrating immune responses: How size, shape and rigidity affect the immunogenicity of particulate vaccines. J Control Rel.

[13] Salvador A, Igartua M, Hernández RM, Pedraz JL (2011). An Overview on the Field of Micro- and Nanotechnologies for Synthetic Peptide-Based Vaccines. J Drug Deliv..

[14] Vartak A, Sucheck SJ (2016). Recent advances in subunit vaccine carriers. Vaccines.

[15] Silva AL, Soema PC, Slütter B, Ossendorp F, Jiskoot W (2016). PLGA particulate delivery systems for subunit vaccines: Linking particle properties to immunogenicity. Human Vacc Immunother.

[16] Makadia HK, Siegel SJ (2011). Poly Lactic-co-Glycolic Acid (PLGA) as biodegradable controlled drug delivery carrier. Polymers (Basel)..

[17] Koerner J, Horvath D, Groettrup M (2019). Harnessing Dendritic Cells for Poly (D, L-lactide-co-glycolide) Microspheres (PLGA MS)-Mediated Anti-tumor Therapy. Front Immunol..

[18] Shima F, Uto T, Akagi T, Baba M, Akashi M (2013). Size effect of amphiphilic poly(γ-glutamic acid) nanoparticles on cellular uptake and maturation of dendritic cells in vivo. Acta Biomater..

[19] Ottenhoff THM, Kaufmann SHE (2012). Vaccines against tuberculosis: Where are we and where do we need to go?. PLoS Pathog.

[20] Christensen D, Korsholm KS, Andersen P, Agger EM (2011). Cationic liposomes as vaccine adjuvants. Expert Rev Vaccines.

[21] Hafner AM, Corthésy B, Merkle HP (2013). Particulate formulations for the delivery of poly(I: C) as vaccine adjuvant. Adv Drug Del Rev..

[22] Duthie MS, Windish HP, Fox CB, Reed SG (2011). Use of defined TLR ligands as adjuvants within human vaccines. Immunol Rev..

[23] Wischke C, Zimmermann J, Wessinger B, Schendler A, Borchert HH, Peters JH (2009). Poly(I:C) coated PLGA microparticles induce dendritic cell maturation. Int J Pharm..

[24] Hamdy S, Elamanchili P, Alshamsan A, Molavi O, Satou T, Samuel J (2007). Enhanced antigen-specific primary CD4+ and CD8+ responses by codelivery of ovalbumin and toll-like receptor ligand monophosphoryl lipid A in poly(D, L-lactic-co-glycolic acid) nanoparticles. J Biomed Mater Res A..

[25] Diwan M, Tafaghodi M, Samuel J (2002). Enhancement of immune responses by co-delivery of a CpG oligodeoxynucleotide and tetanus toxoid in biodegradable nanospheres. J Control Release..

[26] Wille-Reece U, Wu CY, Flynn BJ, Kedl RM, Seder RA (2005). Immunization with HIV-1 Gag protein conjugated to a TLR7/8 agonist results in the generation of HIV-1 Gag-specific Th1 and CD8+ T cell responses. J Immunol.

[27] Zhao K, Wang H, Wu C (2011). The immune responses of HLA-A*0201 restricted SARS-CoV S peptide-specific CD8+ T cells are augmented in varying degrees by CpG ODN, PolyI: C and R848. Vaccine..

[28] Roces CB, Christensen D, Perrie Y (2020). Translating the fabrication of protein-loaded poly(lactic-co-glycolic acid) nanoparticles from bench to scale-independent production using microfluidics. Drug Deliv Transl Res..

[29] Yu B, Lee RJ, Lee LJ (2009). Microfluidic Methods for Production of Liposomes. Methods Enzymol.

[30] Sah E, Sah H (2015). Recent trends in preparation of poly(lactide-co-glycolide) nanoparticles by mixing polymeric organic solution with antisolvent. J Nanomater.

[31] Ahn J, Ko J, Lee S, Yu J, Kim YT, Jeon NL (2018). Microfluidics in nanoparticle drug delivery; From synthesis to pre-clinical screening. Adv Drug Del Rev.

[32] Li X, Jiang X (2018). Microfluidics for producing poly (lactic-co-glycolic acid)-based pharmaceutical nanoparticles. Adv Drug Del Rev.

[33] Zhao C, Ge Z, Yang C (2017). Microfluidic techniques for analytes concentration. Micromachines (Basel).

[34] Jahn A, Reiner JE, Vreeland WN, DeVoe DL, Locascio LE, Gaitan M (2008). Preparation of nanoparticles by continuous-flow microfluidics. J Nanopart Res.

[35] Lee J, van der Maaden K, Gooris G, O’Mahony C, Jiskoot W, Bouwstra J. Engineering of an automated nano-droplet dispensing system for fabrication of antigen-loaded dissolving microneedle arrays. Int J Pharm [Internet]. 2021 May 1 [cited 2021 Mar 29];600:120473. Available from: http://www.ncbi.nlm.nih.gov/pubmed/33737094. 10.1016/j.ijpharm.2021.120473 33737094

[36] Leone M, Priester MI, Romeijn S, Nejadnik MR, Mönkäre J, O’Mahony C (2019). Hyaluronan-based dissolving microneedles with high antigen content for intradermal vaccination: formulation, physicochemical characterization and immunogenicity assessment. Eur J Pharm Biopharm.

[37] Tian Y, Lee J, van der Maaden K, Bhide Y, de Vries-Idema JJ, Akkerman R, et al. Intradermal administration of influenza vaccine with trehalose and pullulan-based dissolving microneedle arrays. J Pharm Sci [Internet]. 2022 Apr 1 [cited 2022 Mar 31];111(4):1070–80. Available from: http://www.ncbi.nlm.nih.gov/pubmed/35122832. 10.1016/j.xphs.2022.01.033 35122832

[38] Benne N, Leboux RJT, Glandrup M, van Duijn J, Lozano Vigario F, Neustrup MA, et al. Atomic force microscopy measurements of anionic liposomes reveal the effect of liposomal rigidity on antigen-specific regulatory T cell responses. J Control Release [Internet]. 2020 Feb 1 [cited 2023 Oct 17];318:246–55. Available from: https://pubmed.ncbi.nlm.nih.gov/31812539/. 10.1016/j.jconrel.2019.12.003 31812539

[39] Immunology: Ovalbumin (OVA) Challenge [Internet]. Available from: https://www.taconic.com/find-your-model/gems/cryopreserved-models/knockout-repository/phenotypic-data-packages/comprehensive/ovalbumin-challenge.html.

[40] Shi S, Zhu H, Xia X, Liang Z, Ma X, Sun B (2019). Vaccine adjuvants: Understanding the structure and mechanism of adjuvanticity. Vaccine..

[41] Benne N, van Duijn J, Kuiper J, Jiskoot W, Slütter B. Orchestrating immune responses: How size, shape and rigidity affect the immunogenicity of particulate vaccines. J Control Release [Internet]. 2016 Jul 28 [cited 2023 May 10];234:124–34. Available from: https://pubmed.ncbi.nlm.nih.gov/27221070/. 10.1016/j.jconrel.2016.05.033 27221070

[42] Mönkäre J, Pontier M, van Kampen EEM, Du G, Leone M, Romeijn S (2018). Development of PLGA nanoparticle loaded dissolving microneedles and comparison with hollow microneedles in intradermal vaccine delivery. Eur J Pharm Biopharm.

[43] Hajavi J, Ebrahimian M, Sankian M, Khakzad MR, Hashemi M (2018). Optimization of PLGA formulation containing protein or peptide-based antigen: Recent advances. J Biomed Mater Res - Part A..

[44] Sahoo SK, Panyam J, Prabha S, Labhasetwar V (2002). Residual polyvinyl alcohol associated with poly (D, L-lactide-co-glycolide) nanoparticles affects their physical properties and cellular uptake. J Control Release..

[45] McGrath MG, Vucen S, Vrdoljak A, Kelly A, O’Mahony C, Crean AM (2014). Production of dissolvable microneedles using an atomised spray process: Effect of microneedle composition on skin penetration. Eur J Pharm Biopharm.

[46] Kim JD, Kim M, Yang H, Lee K, Jung H. Droplet-born air blowing: novel dissolving microneedle fabrication. J Control Release [Internet]. 2013 Sep 28 [cited 2022 Sep 1];170(3):430–6. Available from: http://www.ncbi.nlm.nih.gov/pubmed/23742882. 10.1016/j.jconrel.2013.05.026 23742882

