# Supplementary material for: Intradermal Vaccination with PLGA Nanoparticles via Dissolving Microneedles and Classical Injection Needles
Source: Pharm Res. 2024 Feb 8;41(2):305–19. doi: 10.1007/s11095-024-03665-7 (PMC10879229; doi:10.1007/s11095-024-03665-7)
Supplement: Supplementary file 1 — Supplementary file1 (DOCX 10604 KB) [file 11095_2024_3665_MOESM1_ESM.docx]

**Supplementary material**

**
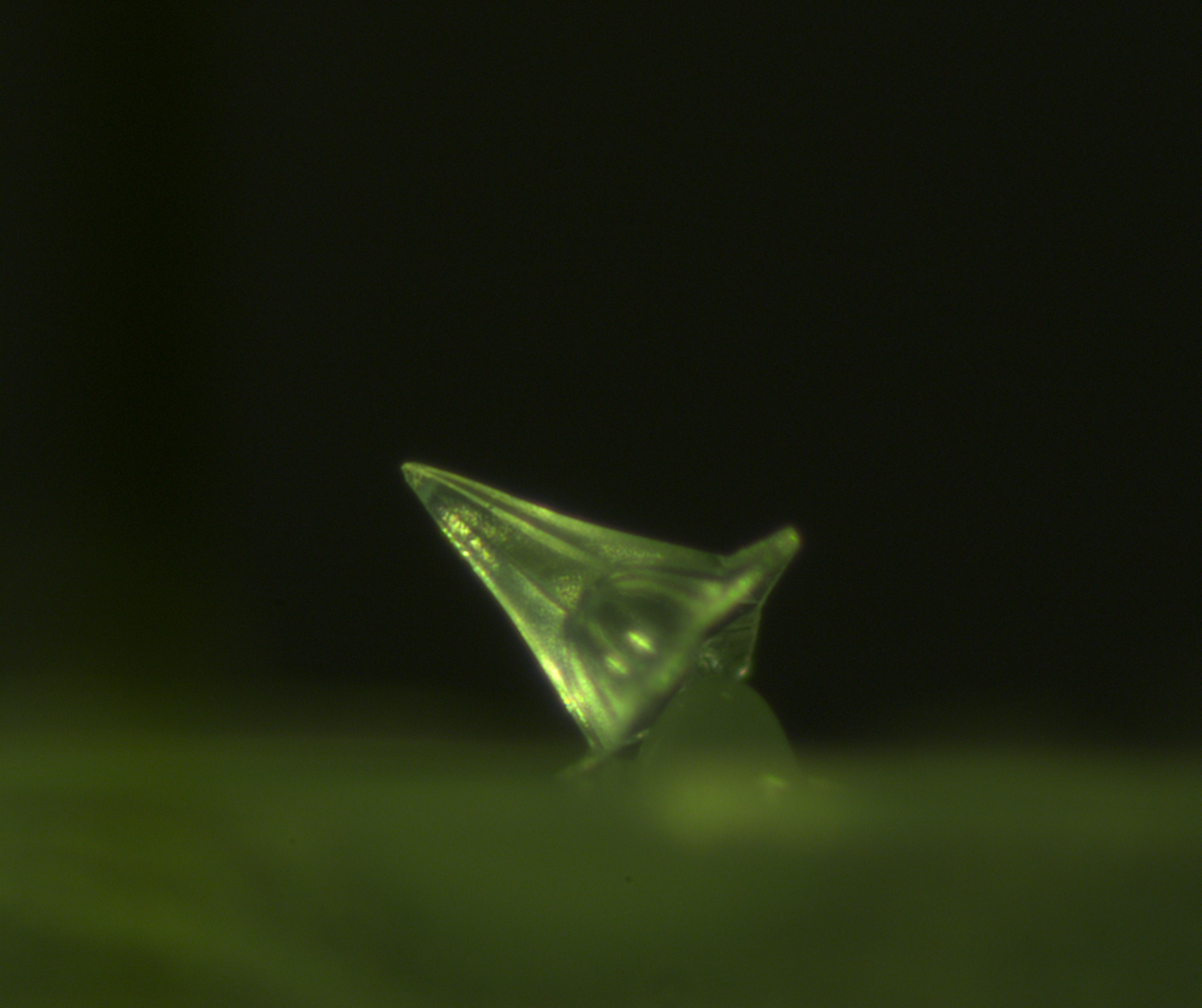
**

Figure S1. Separated microneedle from the array. All nine microneedle was separated from the array using blade to quantify the loading of OVA/CpG.

(a)


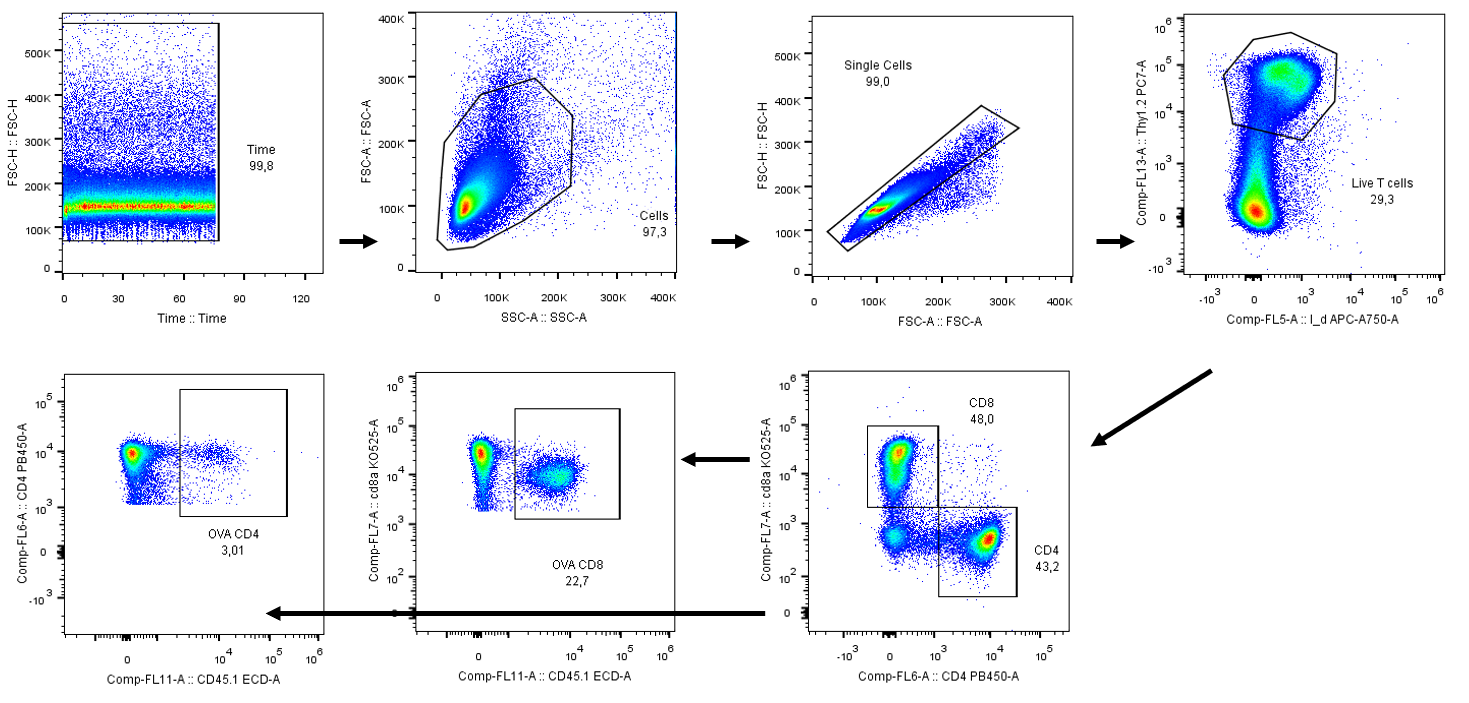


 (b)


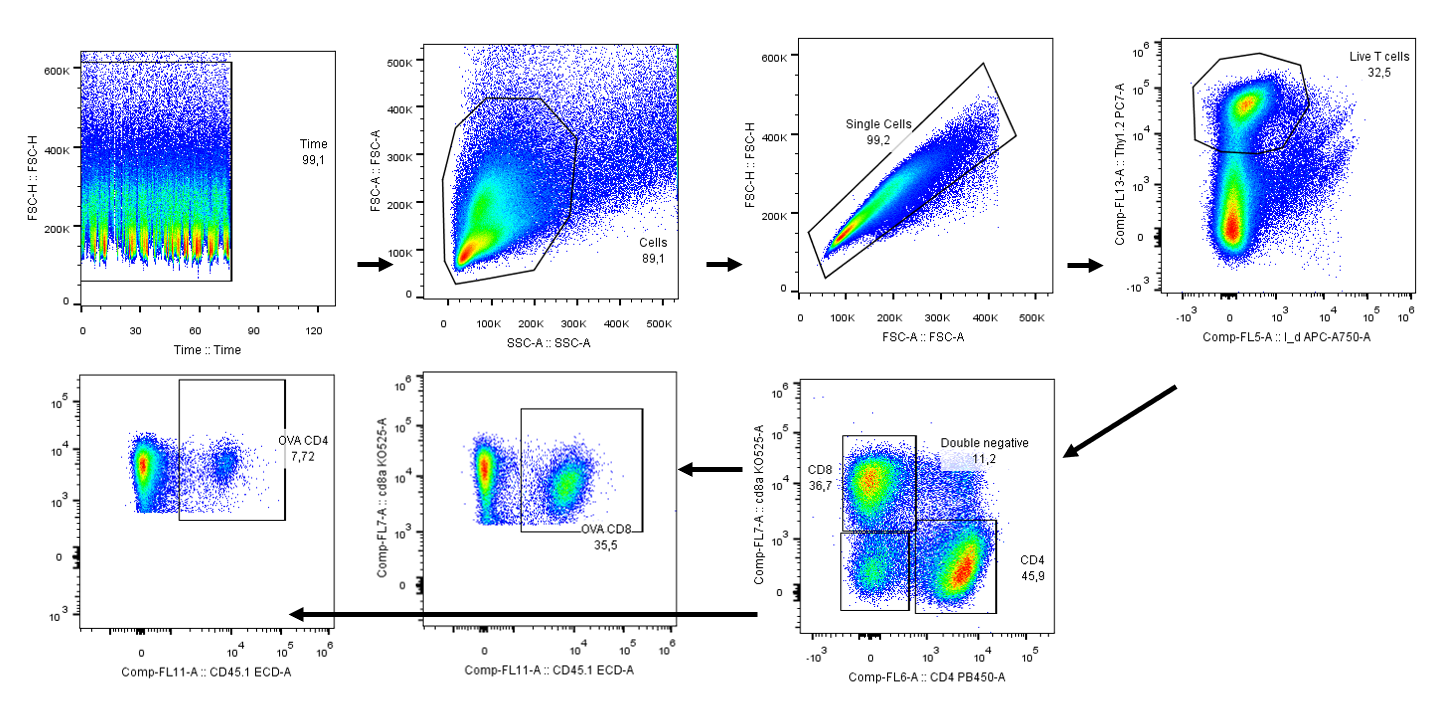


Figure S2. The gating strategy of (a) blood cells and (b) spleen cells for the OVA-specific T-cell responses. First the cells were gated, then single cells, followed by live T cells, CD4^+^ and CD8^+^ cells, and then OVA-specific T cells.

**
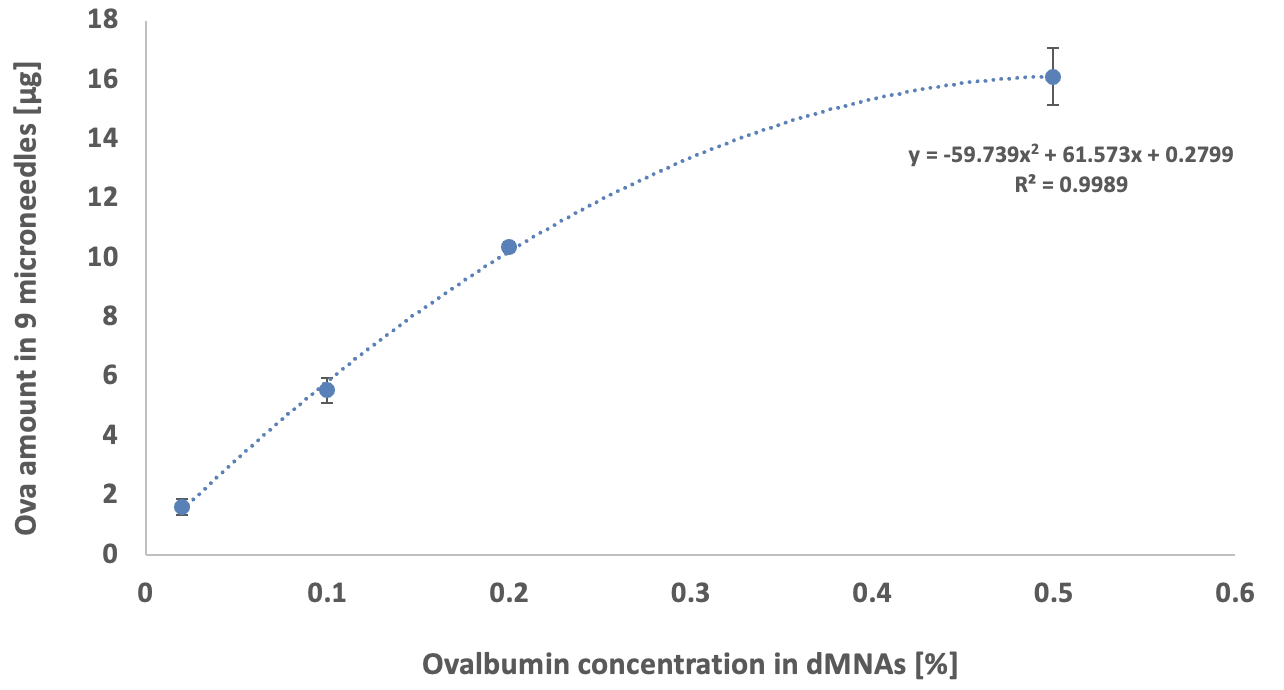
**

**Figure S3.** OVA amount in nine microneedles of 0.02%, 0.1%, 0.2%, 0.5% (w/v) OVA loaded dMNAs


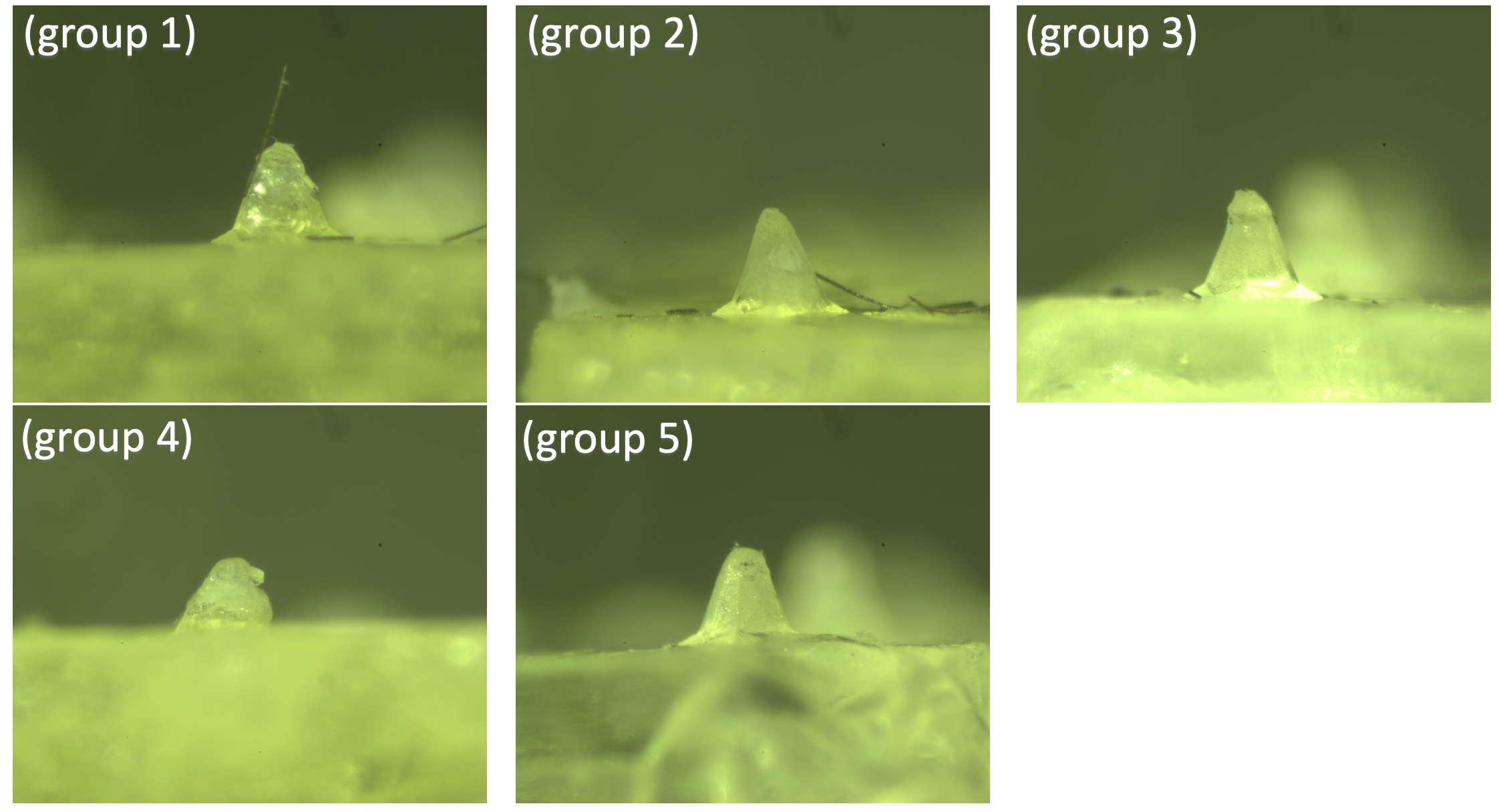


**Figure S4.** Leftover dMNAs and microneedles after vaccination


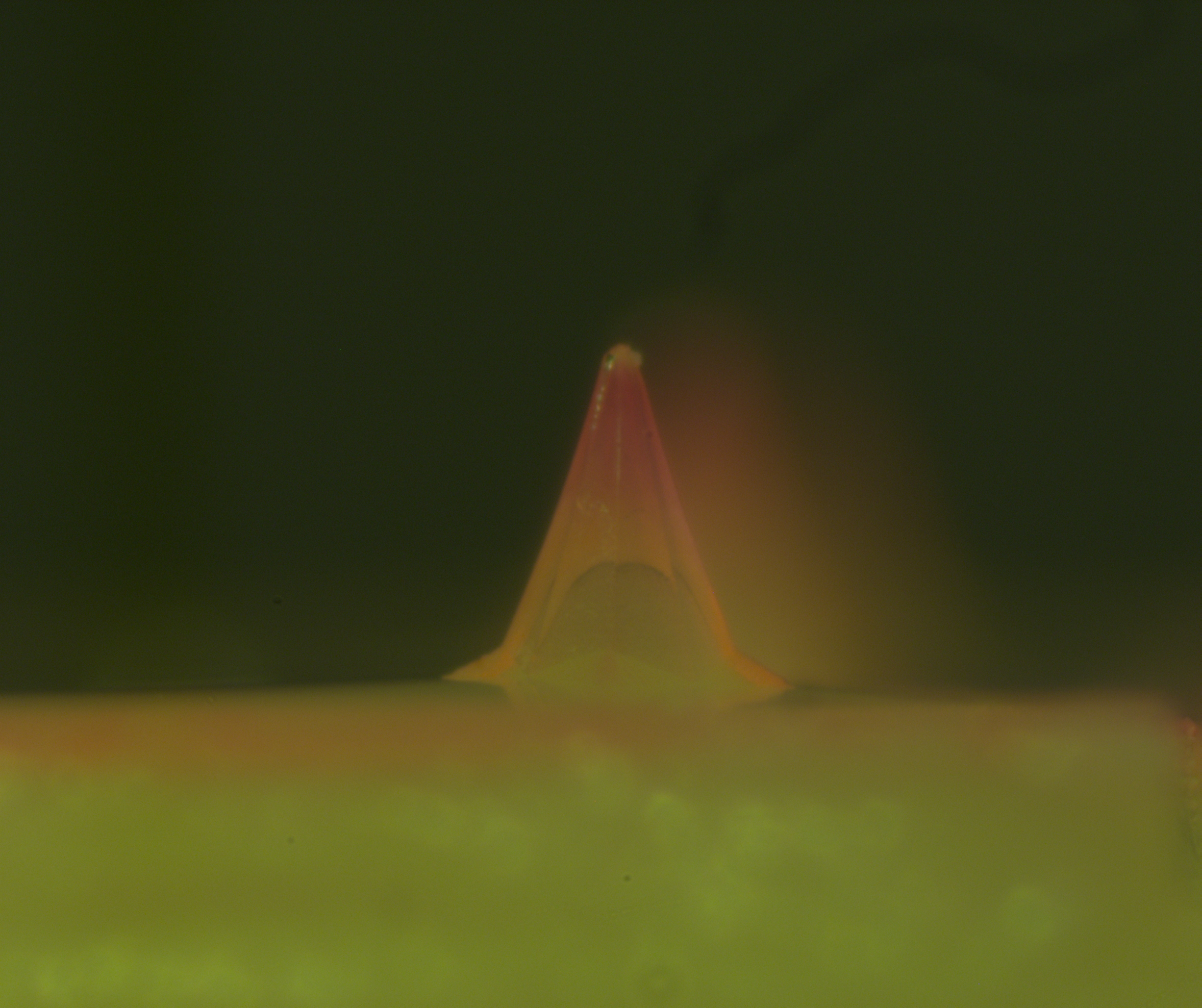


**Figure S5.** Rhodamine b labelled PLGA NPs loaded PVA microneedle. The weight ratio between PLGA NPs and PVA is 1:4, and centrifugal force make PLGA NPs concentrated to the tip of the microneedle.
